# Training needs and perspectives of community health workers in relation to integrating child mental health care into primary health care in a rural setting in sub-Saharan Africa: a mixed methods study

**DOI:** 10.1186/s13033-017-0121-y

**Published:** 2017-02-01

**Authors:** Dejene Tilahun, Charlotte Hanlon, Mesfin Araya, Basiro Davey, Rosa A. Hoekstra, Abebaw Fekadu

**Affiliations:** 1 Department of Psychiatry, School of Medicine, College of Health Sciences, Addis Ababa University, PO 9086, Addis Ababa, Ethiopia; 2 Department of Health Education and Behavioural Sciences, College of Public Health and Medical Science, Jimma University, Jimma, Ethiopia; 3 Health Services and Population Research Department, Institute of Psychiatry, Psychology and Neuroscience, Centre for Global Mental Health, King’s College London, London, UK; 4 Department of Life, Health and Chemical Sciences, The Open University, Milton Keynes, UK; 5 Department of Psychology, Institute of Psychiatry, Psychology and Neuroscience, King’s College London, London, UK; 6 Centre for Innovative Drug Development and Therapeutic Trials for Africa, College of Health Sciences, Addis Ababa University, Addis Ababa, Ethiopia; 7 Department of Psychological Medicine, Institute of Psychiatry, Psychology and Neuroscience, Centre for Affective Disorders, King’s College London, London, UK

**Keywords:** Developmental disabilities, Community health workers, Primary health care, Developing countries, Africa, Ethiopia

## Abstract

**Background:**

Community health workers can help to address the substantial unmet need for child mental health care in low and middle income countries. However, little is known about their training needs for this potential role. The aim of this study was to examine training needs and perspectives of community health extension workers (HEWs) in relation to providing child mental health care in rural Ethiopia.

**Methods:**

The study was conducted in the Southern Nations, Nationalities and Peoples’ Region of Ethiopia. A mixed methods approach was used. A total of 104 HEWs who had received training in child mental health using the Health Education and Training (HEAT) curriculum were interviewed using a structured survey. In-depth interviews were then conducted with 11 HEWs purposively selected on the basis of the administrative zone they had come from. A framework approach was used for qualitative data analysis.

**Results:**

Most of the HEWs (88.5%; n = 93/104) reported that they were interested in the training provided and all respondents considered child mental health to be important. The perceived benefits of training included improved knowledge (n = 52), case identification (n = 14) and service provision (n = 22). While most of the participants had their training four months prior to the interview, over a third of them (35.6%; n = 37) had already organized mental health awareness-raising meetings. Participants in the qualitative interviews considered the problem of child mental disorders to be widespread and to cause a large burden to the family and the affected children. They reported that improving their competence and knowledge was important to address the problem and to tackle stigma and discrimination. Participants also listed some barriers for service provision, including lack of competence, stigma and institutional constraints. Opportunities mentioned included staff commitment, high levels of interest and a positive attitude towards providing the service.

**Conclusions:**

Although the HEAT training on child mental health was brief, it appears to have had some impact in improving knowledge and care provision. If the key barriers to service provision are addressed and supported by policy guidance, community health workers may contribute substantially in addressing the treatment gap for children with mental health needs.

## Background

Globally, 10 to 20% of children are estimated to have mental health problems [1, 2]. In Ethiopia, childhood mental problems have been found to affect 12–25% of children [3–5]; these problems are found to be associated with disability, cost and poorer quality of life of children and their families [1, 2, 6–8]. Moreover, the treatment gap approaches 90% in low and middle income countries (LMIC), largely due to lack of detection of these problems and a severe shortage of trained manpower [9, 10].

Community health extension workers (HEWs) have the potential to play a major role in addressing the treatment gap in LMIC [6, 11, 12]. There is clear evidence of the beneficial role of community health workers in promoting immunization uptake, improving outcomes for acute respiratory infections and malaria care in LMIC [13–16]. There is also evidence that identification and treatment of mental disorders may be improved through training of HEWs [9, 17–19]. Delivery of integrated care at the primary health care (PHC) level supported by HEWs may even reduce stigma and discrimination [17, 19–21]. However, at present there is insufficient evidence to justify recommendations for the deployment of community health workers for child mental health care.

In Ethiopia, the health extension program was launched in 2003 [22]. Since that time, around 38,000 HEWs have been deployed to rural areas across the country. HEWs are mostly female health workers tasked mainly with providing promotive, preventive and rehabilitative care after one year of training. More specifically, HEWs in Ethiopia promote immunization uptake, antenatal care and prevention of malaria, both at their health post and through outreach services by visiting the houses of people in their community [23, 24]. Evaluation of the health extension programme in Ethiopia has indicated some notable successes, including a higher proportion of children vaccinated against communicable diseases and an increase in the use of bed nets to prevent malaria [25]. However, HEWs have not been involved in the care of children with mental health problems in Ethiopia. Additionally, the initial HEW training programme did not include any training in mental health. To address these concerns, in 2011 the Federal Ministry of Health (FMOH) of Ethiopia, in partnership with the Open University UK, developed a new training programme to upgrade HEWs (to level 4 or diploma grade), which included training in mental health through the Health Education and Training (HEAT) programme. The HEAT curriculum covers key topics in child and maternal health, family planning and sexual health, environmental hygiene, communicable and non-communicable diseases. HEAT is delivered primarily through face-to-face teaching (lecture methods) supported by printed training modules for the HEWs. The mental health section comprises ten study sessions, equivalent to two weeks of fulltime study. One study session, (i.e. equivalent to one day study time) focuses on child mental health, covering topics such as the importance of healthy development, enuresis and intellectual disability, with a brief mention of autism. (The HEAT study materials are available in full as open educational resources from the HEAT (Health Education and Training) website at http://www.open.ac.uk/africa/heat).

When this study was initiated, about 1300 HEWs were trained in the HEAT modules in a pilot project across the country and currently 12,700 have completed the HEAT training and have been upgraded to Level-4 (Personal Communication, Federal Ministry of Health, 2016). However, competency needs and perspectives of HEWs about the integration of child mental health care into their routine practice have not previously been explored. Therefore this study examined training needs and perspectives of HEWs, including barriers and facilitators, in relation to integrating child mental health care into community-based PHC services in Ethiopia.

## Methods

### Setting

The study was conducted in the Southern Nations, Nationalities, and Peoples’ Region (SNNPR) of Ethiopia. The SNNPR was chosen because of existing research infrastructure of Addis Ababa University in the region. From the Ethiopian census of 2007 [26], SNNPR has an estimated population of around 15 million and hosts over half of the ethnic diversity of Ethiopia. The region is predominantly rural; nearly 90% of inhabitants reside in rural areas. The official language of SNNPR is Amharic. Between 2003 and 2009, SNNPR trained 7492 rural HEWs [27]. In 2011, 208 HEWs from SNNPR enrolled in the HEAT training, of which 204 successfully completed the training.

### Study design

A mixed methods approach, consisting of a cross-sectional survey and qualitative study, was employed. The qualitative study was included to obtain a more in-depth understanding of the experiences and perspectives of HEWs.

### Participants and sampling

A total of 204 HEWs from the Southern Nations, Nationalities, and Peoples’ Region (SNNPR) were trained in HEAT in 2011. There are four HEAT training centers in the region. Our study sample was recruited from the 116 HEWs trained in the Hawassa and Hossana facilities. All HEWs are female due to the policy of the Federal Ministry of Health of Ethiopia.

The HEAT trained HEWs were identified via the regional health bureau and were informed in advance about the study. If the HEW agreed to participate in the study, the HEWs were invited to the nearest College of Health Sciences (Hawasa and Hosana) to carry out the assessments centrally. All 116 HEAT trained HEWs were invited and 104 (89.7%) attended. All of those who attended gave informed consent to participate.

For the qualitative study, in-depth interviews were conducted with 11 HEAT-trained HEWs selected purposively on the basis of the administrative zone within which they worked. This “zone-based” selection was to ensure diversity of localities of origin in order to capture the broader range of experiences.

### Measures

Both structured and open-ended questions were used. The structured questionnaire comprised two parts: socio-demographic characteristics (age, religion, educational status and work experience) and learning needs and experience. The learning needs and experience questionnaire gauged the interest of participants on attending the training on child mental health and on their perspectives regarding the quality of training resources and the importance of the topic. This section also included questions on whether or not the participants had made use of the training resources in their practice, for example, whether they had organized a mental health awareness meeting in the community to discuss about mental health problems. The open-ended questionnaire explored whether the study material helped in their daily practice, and solicited suggestions on improving the study material, including what the HEWs thought would be useful to include in the training. For in-depth interviews, a topic guide was prepared to guide the discussion with HEWs. The in-depth interviews expanded on the quantitative survey and probed specifically around child developmental disorders, autism and intellectual disability (ID); a specific focus on developmental disorders was chosen because these conditions are explicitly included in WHO’s mhGAP programme as a priority condition for action [28]. The in-depth interviews explored the following areas: training needs and the perceived importance of the training; perspectives on the modules and training; barriers and facilitators to developing child mental health services; and suggestions for improvement of the training materials.

### Data collection

The questionnaires were prepared in English, translated into Amharic and then back-translated into English to ensure consistency. Amharic is the official language of Ethiopia and the region. The instruments were pre-tested in a small group of HEWs. A final version of the questionnaire was established following feedback from the pre-test. All data collectors were women who had completed secondary school and had extensive experience in data collection. The data collectors were thus of the same gender and of similar socio-economic status as the participants, limiting possible bias in their responses. The data collectors were trained to administer the questionnaire through face-to-face interviews. Training was given over five days to ensure that the data collectors were familiar with the data collection procedures, the questionnaire, information sheets and consent forms.

The in-depth interviews were conducted by DT in private in Amharic. With the agreement of participants, the interviews were audio-recorded. Electronic Amharic transcripts were produced and these were translated into English.

### Data analysis

The quantitative data were analysed using SPSS version 20 (IBM SPSS Statistics 20). The analyses were primarily descriptive. Categorical values were summarized in absolute numbers, and percentages were calculated to one decimal place. Mean and standard deviation was employed for continuous variables. For in-depth interview data, analyses utilized the interview transcripts, interview summaries and the field notes. The English transcripts were analyzed using OpenCode 3.6 qualitative data analysis software [29]. A framework analysis was used [30], a common approach in qualitative research that employs several stages: familiarization, identifying the thematic framework, indexing, charting and interpretation. The first author coded all of the transcripts and one of the authors (AF) read and gave feedback on the translations, and coding process for possible themes/codes and quotes. An initial list of codes and themes was developed and reviewed and then refined after further re-reading. Illustrative quotes were selected for the resulting themes. The quotes were sufficiently in-depth to allow readers to understand the context of the responses. Finally, the analyzed qualitative data were triangulated together with the quantitative findings.

## Results

### Socio-demographic characteristics of respondents

A total of 104 HEAT trained HEWs were included in the analyses. All HEWs were female and their mean age was 25.8 years (SD 3.5). Most of the participants were either protestant (67.3%; n = 70) or Orthodox christian (19.2%; n = 20) in religion (Table 1). On average the HEWs had been employed for about 76 months at the time of the study. All were surveyed about 4 months after their HEAT training. That is, most HEWs had worked for a couple of years as HEW prior to taking up the HEAT upgrading training. All HEWs had returned to their health post after completion of the HEAT training, to continue their work, now as a higher qualified health worker.

**Table 1:** Background characteristics of participating health extension workers (n = 104)

Socio-demographic characteristics	Frequency	Percentage
*Religious affiliation*		
Muslim	12	11.6
Orthodox christian	20	19.2
Catholic	2	1.9
Protestant	70	67.3
*Highest grade you completed at school*		
Grade 10	69	66.3
Grade 12	35	33.7

	Mean (standard deviation)
Age in years	25.8 ± 3.5
Overall work experience as HEW (months)	75.7 ± 13.6
Work experience in current post (months)	69.7 ± 20.1

### Learning needs of HEWs

All HEWs surveyed considered the topic of child mental health to be important or very important (n = 104). Most of the HEAT-trained HEWs (88.5%; n = 93) reported that they were interested in the study modules focusing on childhood mental health problems (Table 2). The perceived quality of all mental health study texts (i.e., not only child mental health) was average or above average (76.9%, n = 80) with 23.1%, n = 24 perceiving it to be below average. Nineteen HEWs (18.3%) did not have access to the study materials. Most of these (53%) who did not have access to the study materials rated the materials as below average while only 16% of those who had the study materials rated the materials as below average.

**Table 2:** Attitudes of Health extension workers towards training and training materials

Questionnaire items	Frequency	Percentage
*Access to training materials*		
Yes	85	81.7
No	19	18.3
*Opinion of quality of study material*		
Extremely poor	14	13.5
Below average	10	9.6
Average	33	31.7
Above average	17	16.4
Excellent	30	28.8
*Overall, did you find the mental health and childhood problems study text interesting to study?*		
Not at all interested	5	4.8
Of little interest	7	6.7
Moderately interesting	35	33.7
Interesting	18	17.3
Very interesting	39	37.5
*Do you feel that the topic of childhood behavioural and developmental problems is important for a HEW?*		
Not at all important	0	0
Of little importance	0	0
Moderately important	0	0
Important	4	3.8
Very important	100	96.2

The in-depth interviews largely reinforced the above views. However, some participants expressed that important concepts about child mental health problems, especially on child developmental disorders, were not covered adequately.

*“[…] I think there is nothing missing from the contents of the modules on adult mental disorders. However, training on mental health needs of children lacked many things. The module […] covered very little on child developmental disorders and autism. It would be better to include the symptoms of developmental disorders, including autism, and details on how to make diagnosis, identify causes and provide treatment. The other problem was that the original teaching materials [on mental health] were not available and we had to make copies [for our use].” (HEW 5).*

Regarding the importance of the subject of child mental health problems, one participant stated:

*“[…] Generally the training was very important; I gained knowledge about developmental disorders…When I compare myself from previous, I learned many things […] after training I learned how to treat, identify and manage childhood mental problems.” (HEW 1).*

### Experience and role of HEWs

Nearly half of the trained HEWs (46.1%; n = 48) reported that they made use of the mental health training modules, which included the child mental health section, once or twice a week (Table 3). The in-depth interviews revealed some challenges in their experience of using the training materials.

**Table 3:** Experience of health extension workers in utilization of the training materials

Questionnaire items	Frequency	Percentage
*Do you use any of the training provided in the mental health and childhood problems study material in your work?*		
No, never	14	13.5
Yes, but only rarely (once or twice a year)	19	18.3
Yes, sometimes (about once a month)	23	22.1
Yes, often (about once a week)	20	19.2
Yes, very often (more than once a week)	28	26.9
*If Yes, how did the training help you in your daily work?* ^a^		
Increasing knowledge and awareness	52	50.0
Improve the service	22	21.2
Identification of the case	14	13.5
Motivated me to do and read the materials	8	7.7
Counseling	8	7.7
Referral	6	5.8
*Have you organized a mental health awareness meeting in your community?*		
No, never	67	64.4
Yes, but only once	26	25.0
Yes, two or more meeting	11	10.6

^a^ Respondents could provide multiple answers

*“[…] The instruction in the English language was difficult to understand […] In addition, as this was a new course, my limited previous training and knowledge about child developmental disorder made it very difficult to understand child mental disorders.” (HEW 1).*

The other participants also emphasized the problem:

*“There is a language problem […]. Even the few things we understood about adult mental problems was because the instructor translated his lecture into Amharic [the local language] during his teaching. The child mental health session was not clear and has only limited content with very little [information] on autism. So we need to learn in detail about each kind of the disorder, using simple language.” (HEW 2).*

When the respondents were asked about how the training and training materials helped in their daily work, most reported benefits as improvement in knowledge and awareness, case identification and provision of service (Table 3). Qualitative study participants confirmed similar benefits of training: as a gain in knowledge, skills, a positive attitude and confidence. For example, one participant stated:

*“… After training I gained knowledge, [was] able to detect child mental disorders, including autism. This motivated me to give health education to raise awareness in the community about misconceptions, [for example] not to hide a child with developmental disorder, negative attitudes, and stigma and was able to refer cases to the hospital and provide advice on care and support.” (HEW 9).*

On the other hand, many of the participants reported having poor knowledge and said that they were unable to distinguish autism from the other types of child mental disorders. They also lacked the skills and confidence to identify, treat and refer child mental health problems. Most also said they were fearful and embarrassed about approaching and helping families with an affected child and that their negative attitude might have been a barrier towards providing support to children with development problems.

*“[…] the other problems that affected me doing this work were my own fear and misperception that the family may not accept my advice and my belief that such cases could not be cured or have treatment.” (HEW 3).*

*“[…] my negative attitude and misperception towards improvement of children with developmental disorder is a problem towards helping a child with mental problems. (HEW 9).*

Even though the HEWs had only returned to their community 4 months prior to the survey, about one-third (35.6%, n = 37) of HEWs reported that they had organized a mental health awareness meeting in their community since (Table 3). However, other participants reported little activity at their work place because of their poor competency and health institution constraints such as lack of knowledge and skill of PHC workers at health centers, large coverage area and overload of work, problem of transport, lack of supportive supervision and coordination. The activities the HEWs reported that they had performed included identification of cases, counselling, awareness raising, organizing community meetings, provision of direct services, referring and follow up and monitoring during home visits.

### Rationale for training

All HEWs reported a high level of need for training on identification and treatment of child mental health problems. Most of surveyed HEWs said that child mental problems were very common in the community and had a large impact on the family and individual. The impacts were mostly perceived to be related to financial, social and health related burdens. The social burden mentioned by the participants were related to the child’s behavioural difficulties, carers being unable to work and being limited in their ability to participate in social activities because of needing to be the carer, negative attitudes from the community and stigma. Economic burden on families, such as the cost of transport and treatment was also reported by HEWs. Most of the HEWs interviewed in-depth thought that the caregivers’ burdens were aggravated by the lack of availability of treatment at PHC and poor awareness, negative attitudes and stigma from the community about the condition.

*“[…] there are many children with developmental disorder in the community; for example in my kebele [administrative sub*-*district, each consisting of about 5000 people] I have identified four cases, all of the children do not speak even when they are hungry […]. I gave advice on hygiene and appropriate diet to the families of children with such problems […].”(*HEW 8*).*

*“[…] Financial problems of the family to access treatment for their child with a developmental disorder after referral was a challenge that hindered my day to day work […] other problems are lack of available services and resources….for example special schools to educate these children….and lack of trained health personnel to help children and their families at health centers [the primary care facility to which HEWs would refer] in rural area. These are the most important problems. So treatment, care and support need to be found and improved at the health center level […].” (HEW 4).*

*“[…] the other problem was negative attitudes and misperceptions in the community and family about treatment and the cause [of their child’s condition] during counselling and awareness raising … i.e. the belief of the community that these kinds of conditions do not need modern treatment and can be transmitted to others. The belief is that the cause is a punishment from God, and that intervention is only possible through traditional or religious treatments such as praying, holy water, spiritual beliefs* etc*. […].” (HEW* 5*).*

The HEWs also reported a need for further training due to their low level of baseline competency in the identification of affected children, to provide interventions and awareness of referral services. They recognized a need to improve motivation across all HEWs to provide this service. They also recognized a need to improve the quality of care and safety of families and their affected child. The majority of HEWs reported that, as they were already delivering different packages of home-based care for mothers and children, they would easily be able to identify and treat the children if they acquired adequate knowledge and skills.

HEWs reported the need for expanded in-service training. When asked to suggest specific content to be included in future training on mental health and childhood problems, the most commonly reported training need was for detailed, clear and separate training on child developmental disorders (74.1%, n = 77) (Table 4). Almost all of the in-depth interview participants wanted in-service training, especially in the identification, causes, prevention and treatment of child mental health problems, including developmental disorders.

**Table 4:** Self-reported training needs of health extension workers

Health extension worker recommendations	Frequency	Percentage
*Suggested area to be included in mental health and childhood problems study text* ^a^		
Detailed, clear and separate session	77	74.1
Signs, symptoms and cause or detail on identification	20	19.2
Management and treatment	16	15.4
More practical part i.e. about first aid	11	10.6
*Suggestions on how study text can be improved* ^a^		
Detailed, clear and separate module	77	74.0
Practical training	22	21.2
Simple Amharic	7	6.7
Receiving module on time	8	7.7
Adequate time for training	6	5.8

^a^ Respondents could provide multiple answers

The most important unmet need on methods of training was preparation of a detailed, clear and separate module on child mental health and developmental disorders (74.0%, n = 77) and practical training mostly on how to give support, including first aid to families with mental health needs (21.2%, n = 22) (Table 4). In-depth interviews also indicated similar training needs. One HEW said;

*“We are giving service at the grass root level and in the community, so we need to be up to date with the new information and advance in our knowledge and skill to ensure detection, treatment and referral. Then we can share and provide an appropriate service for model family and community/family [1 out of 6 families are trained as ‘model’ families for health promotion and education activities] but we didn’t get this opportunity. […] my suggestions as a means of improvements are refresher courses (in*-*service training) […] about developmental disorders.” (HEW 7).*

### Barriers to integration of child mental health care

Many barriers were reported to affect the integration of child mental health care into existing routines. The identified barriers were at the level of the individual, community and institution.

The barriers at the level of the HEW included poor knowledge and skills, negative attitudes and stigma and demotivation. The barriers at the community level were misconceptions, negative attitudes, stigma and discrimination. This is stated by one participant as quoted below;

***“*** *[…] lack of adequate knowledge and skill on child developmental disorders also minimize my confidence in helping families. Negative attitude and misperception held by the community and families also decrease our motivation to help […].” (HEW 5).*

Two HEWs described how disease attributions may be barriers encountered within a family:

*I have got one child in our survey; he does not talk. His parents were hiding information about him. They thought that this type of disease is cured through traditional or spiritual means. They said [his illness was] due to spirit possession*-*likift*-*because someone had given him some potion. When I saw the child he was very pale and […] chained. Then I started to discuss how this child should be treated […] but parents said if this child got medical treatment he may die; instead traditional treatment like slaughtering [an animal] and praying were their preferred treatment. Finally we planned another appointment for further intervention and discussion about referral of the child.” [HEW 2].*

*“[…] at the beginning he (father) became resistant because of his strong belief that [the child’s] condition was not curable through modern treatment because it was the result of punishment from God and that this child could only be treated by holy water.” [HEW 3].*

In addition, low expectation of community and family of input from HEWs, and lack of appreciation from the community leading to demotivation of HEWs, were also mentioned as barriers.

Institutional barriers such as constraints of resources, including lack of trained health professionals at health centers, lack of services and facilities and financial problems for transport, treatment and other costs, were common barriers mentioned by HEWs. Minimal attention by stakeholders or government bodies, lack of supportive supervision, coordination and collaborative work were also mentioned as constraints. The other barriers for integration of the mental health service into routine practice were insufficient training due to inadequate time allocation, content, methods and scope of training, minimal attention on mental health by tutors, inaccessibility of the module and other reference materials, and heavy work load.

*“[…] lack of health services (treatment, care and support) and special schools to help these children and their families in the rural areas are the most important barriers. Constraints of time because of overload of work are also another barrier. So care and support including treatment need to be improved.” (HEW 4).*

Other HEWs also explained that when there are no skilled professionals to provide care at the health centers, or a family cannot afford to take their child to a health centre, this limited the acceptability of the care they can provide.

***“*** *Lack of skilled health professionals to help children at the health center after referral and financial problems of parents to take up referral also reduces our acceptability.” (HEW 5).*

*“Ignorance of the community and lack of attention from the government are also some of the problems that needed to be solved.” (HEW 9).*

### Facilitating factors to overcome barriers for integrating child mental health care

Most respondents considered child mental health problems as important and had a positive attitude and interest toward training and providing care for children with mental health problems. The other facilitating factors reported were availability and the use of reference materials provided as part of the HEAT training. High motivation and willingness of HEWs to apply and maintain an effort in their task, positive attitude and suggestion of HEWs to integrate the child mental health care into routine general health service were also mentioned as facilitating factors for integrating child mental health service. Some HEWs were identifying child mental problems and getting involved in the actual service delivery of child mental health care. One participant explained the benefits as:

*“There is a change in my knowledge and skill and the training helped me to plan and to survey for detection or diagnosis of cases. It also helped me in mobilizing the local community; this supports the detection of cases because people know where children with mental problems including autism are found i.e. they [community] can easily identify in which house a child with autism is found.” (HEW 2).*

The organizational structure of the district and the work of HEWs with families was considered as an important facilitator.

*“[…] Working on child mental health problems is not problematic or a challenge, because our work is mostly with mothers; so we can easily access children with no [extra work] overload. This work is very important because children are the future generation of the country.” (HEW 7).*

HEWs also suggested many facilitators to overcome barriers for integrating child mental health care. The suggested facilitators for addressing the barriers in performing tasks and integrating child mental health care services were in-service training for HEWs and general health staff and awareness raising programmes using mass media for those in positions of authority and stakeholders. Expanding availability of services in the health sector, school setting and other settings were also suggested for the improvement of care for the child and family. Facilitating referral linkage, coordination of activity, integration and collaboration of work and continuous supportive supervision were also suggested. The other most important suggestions to address barriers were practical exposure to mental health problems during training and improvement of the contents, methods and scope of training. Concerning this issue, one of the HEWs said;

*“Accessibility and availability of information and services for children, their family and the community about developmental disorders and child mental health care are mandatory. In addition in*-*service training for all HEWs that did not receive HEAT training should be given i.e. every HEW should get the chance of learning how to detect, treat and help children with developmental disorders, their families and the community…. my friend (HEW in the same kebele) also needs to get this training [HEAT training] to improve treatment, care and support.” [HEW 4].*

*Another participant said “[…] In order to increase awareness*-*raising, motivation and advocacy on child mental problems, attention needs to be given to integrated work, involvement of all important stakeholders e.g. administrators, religious leaders and other stakeholders. So help and integration from other stakeholders are mandatory to bring change, to solve the problems of the individual, their family and community and prevent further burden.” (HEW 5).*

## Discussion

In this mixed methods study of rural community-based HEWs from Ethiopia with very limited training in the mental health needs of children, the burden of mental disorders among children in the community was considered high. We found a high level of interest and motivation to learn about child mental health and developmental needs of children and to provide services. HEWs reported that, through the training, they would be able to improve quality of care and address the issue of the widespread stigma and discrimination of mental health and developmental problems. Integration of mental health care of children into routine community based primary care practice was acceptable to the HEWs. In addition, several facilitators and barriers for integrating child mental health care were mentioned by HEWs.

### Learning needs and experience

The positive attitudes of HEWs towards training about mental health needs of children and the HEAT study resources may facilitate the implementation and integration of mental health care of children into primary health care. This is consistent with previous findings in LMICs among community health workers that indicated acceptability of training [6, 14] and a positive impact of training on improving knowledge, skills, satisfaction, confidence, motivation and actual performance [14, 17–19, 31–33]. Motivation of health workers is known to be an important factor in the successful implementation of services at the primary health care level [14].

One of the encouraging findings of this study is that even though most HEWs had only recently returned to their community to work as HEW after completing their upgrading training, nearly half of the trained HEWs reported that they had been using the mental health training materials regularly in their practice. This has resulted in self-reported improvements in knowledge, skills and attitudes consistent with reports of improved knowledge, competence and performance among HEWs with the use of guidelines and protocols [14, 31–33]. Interventions to encourage the consistent use of the training materials or improving their utility by developing simpler pocket guides or flow charts may be an important next step. A third (35.6%) of the participants had organized a mental health awareness-raising meeting within four months of training. Given the short time following training to organize mental health awareness meetings and the competing responsibilities HEWs have, organizing these meetings appear to be an important achievement induced by the training.

### Rationale for training need

HEWs considered mental health needs among children to be common and this was their primary justification for perceiving that they needed training. They expressed the view that training of frontline HEWs was important in addressing the treatment gap in children. Such rationales have also been identified elsewhere [34, 35] and speak to the need to include public health arguments about the population level benefits of community interventions. The related issues of family needs were also considered to be important reasons for the training. Childhood health needs are reflections of the broader family need and may impact seriously on the broader family context. Again this is also an important consideration in developing training resources for community providers who interact with families as part of their routine practice. The need for improving knowledge and confidence in case detection, and quality care provision were also mentioned as important rationales for training, consistent with other reports from Ethiopia [23–25, 36, 37] and elsewhere [14, 38].

### Barriers for integration of child mental health care service

In our study poor knowledge and skill were the frequently endorsed barriers for integration of child mental health care into primary care. The majority of the study participants reported that they did not know how to detect, support and prevent child mental health problems as a result of poor competence. This finding is consistent with other reports among HEWs [23, 24] in Ethiopia and community health workers [14, 16] in other LMICs. The HEAT curriculum studied by the HEWs only included one day of study on child mental health and developmental disorders; our participants’ responses indicate the need for further training on this topic. The other barriers were negative attitudes and stigma [37, 39]. Compared to other aspects of their work, the high challenge of child mental health care for HEWs in our study may be related to the low baseline knowledge, the lack of referral services and stigma. Moreover, the broader lack of research and policy guidance may play a part. For example child mental disorders were not included in the implementation of the mental health Gap Action Programme in Ethiopia or other implementation studies, such as the programme for improving mental health care [37, 39]. It is important to develop research evidence such as presented here to support the scale up of mental health care and also address negative attitudes and stigma among HEWs and families [17, 19].

Another barrier mentioned in our study was demotivation as a result of low expectations of the community and other stakeholders from the HEWs. Similarly, unrealistic expectations, for example, the vast majority expecting cure [39], poor planning and underestimation of the effort required, were causes of numerous failures of programmes and damaged the credibility of providers [19, 40]. Institutional level factors, including lack of availability and accessibility of services and facilities, and lack of resources such as time, trained health professionals, and lack of supervision and coordination were also reported to be barriers for integration of the service, in keeping with findings from programmes for other disorders [6, 14, 24, 40].

### Facilitating factors or opportunities for integration of service delivery

The recognition of child mental health problems as important and the overall positive rating of the training materials may facilitate integration of child mental health care into routine primary care. Such factors are identified as important enablers of integration and sustainability of programmes implemented by community health workers [6, 14, 18]. The use of the reference resources provided by the training programme and engagement in organization of community awareness programmes are potential enablers, as were identified elsewhere [6, 14, 33, 41, 42]. However, nearly a fifth of the trainees did not have access to the training materials and this problem was augmented by the language difficulty. This speaks to the need for careful adaptation and piloting of such resources before they become more widely available, as well as to ensure availability of resources to all students after roll-out.

The HEWs reported that their training had improved their knowledge, skill and confidence to detect mental disorders in children and to help family members of children with developmental disorders. This confidence, coupled with the high motivation and willingness of HEWs to apply their training, would be important facilitators of integration into routine practice [6, 42]. The new role of supporting child mental health care is also consistent with the role HEWs felt they had and this perception would also be an important facilitator [14, 35, 43].

### Limitations and strengths of the study

It must be noted that the findings of this study represent only the perceptions of HEWs. We did not study the reflections of children with developmental disorders or their families and other stakeholders. The circumscribed geographic area where the study was conducted also affects generalizability of the findings, although the addition of the qualitative study enhances the relevance of our findings. The HEW program is relatively new to the Ethiopian health care delivery system, which limits the comparisons of the current findings with similar studies conducted earlier. However, the study is the first study in Ethiopia among HEWs on child mental health training and provides relevant information in an area of public health challenge of which very little is known, not just in Ethiopia, but in Africa more widely [44].

## Conclusions

Although the HEAT training on child mental health was brief, it appears to have had an important impact in motivating community health workers and in providing services for children with mental health needs and their family. If the key barriers to service provision are addressed and supported by policy guidance, HEWs may contribute substantially in addressing the treatment gap for children with mental health needs.

## Abbreviations

HEWs: health extension workers
HEAT: health Education and Training (HEAT)
HIC: high-income countries
LMICs: low- and middle-income countries
PHC: primary health care
WHO: World Health Organization
